# Diagnostic accuracy of early cognitive indicators in mild cognitive impairment

**DOI:** 10.1590/1980-57642020dn14-040005

**Published:** 2020-12

**Authors:** Marina Martorelli, Larissa Hartle, Gabriel Coutinho, Daniel Correa Mograbi, Daniel Chaves, Claudia Silberman, Helenice Charchat-Fichman

**Affiliations:** 1 Pontifícia Universidade Católica do Rio de Janeiro – Rio de Janeiro, RJ, Brazil.; 2 Centro Universitário Celso Lisboa – Duque de Caxias, RJ, Brazil.; 3 Università degli Studi di Perugia – Perugia, Italia.; 4 Hospital Caxias D’Or – Duque de Caxias, RJ, Brazil.; 5 Universidade Federal do Rio de Janeiro – Rio de Janeiro, RJ, Brazil.

**Keywords:** cognitive dysfunction, Alzheimer disease, diagnostic, cognition, comprometimento cognitivo leve, doença de Alzheimer, diagnóstico, cognição

## Abstract

The aging of the population leads to an increase in the prevalence of dementia and mild cognitive impairment (MCI). Alzheimer's disease (AD) is the most common cause of dementia. Recent studies highlight the early non-amnestic deficits in AD and MCI. The European Union report shows the importance of thoroughly assessing cognitive aspects that have been poorly evaluated, such as processing speed (PS), which could represent early indicators of cognitive decline.

**Objective:**

To analyze the diagnostic accuracy of PS measures in older adults with MCI, AD, and those who are cognitively-healthy.

**Methods:**

A cross-sectional study was conducted by performing an extensive neuropsychological assessment in three samples: 26 control participants, 22 individuals with MCI, and 21 individuals with AD. Analysis of variance (ANOVA) was employed to test the relationship between dependent variables and the clinical group. *Post hoc* tests (Bonferroni test) were used when a significant ANOVA result was found. Finally, the Receiver Operating Characteristic (ROC) curve for PS measures was performed in older adults with MCI and AD compared with cognitively-healthy older adults.

**Results:**

The results showed that deficits in PS measures can be early indicators of cognitive decline in cases of MCI, even when executive functions (EFs) and functionality are preserved. Conversely, AD *versus* MCI presented differences in PS, EFs, and functionality.

**Conclusions:**

The ROC analyses showed that PS measures had discriminative capacities to differentiate individuals with MCI, AD, and cognitively-healthy older adults.

## RESUMO

O envelhecimento da população leva ao aumento da prevalência de demência e comprometimento cognitivo leve (CCL). A doença de Alzheimer (DA) é a causa mais comum de demência. Estudos recentes destacam os déficits precoces não amnésicos em DA e CCL. O relatório da União Europeia mostra a necessidade de avaliar em maior profundidade aspectos cognitivos que atualmente são negligenciados, como a velocidade de processamento (VP), e que podem representar indicadores precoces de declínio cognitivo.

**Objetivo:**

O objetivo deste estudo foi analisar a acurácia diagnóstica de medidas de VP em idosos com CCL, DA e participantes controles.

**Métodos:**

um estudo transversal foi desenvolvido, no qual realizou-se uma extensa avaliação neuropsicológica em 3 amostras: 26 participantes controles, 22 casos de CCL e 21 DA. A relação entre as variáveis dependentes e o grupo clínico foi testada com uma análise de variância (ANOVA). Se uma ANOVA significativa fosse encontrada, testes post hoc foram utilizados. Por fim, a curva ROC para medidas de VP foi realizada em CCL e DA em comparação com indivíduos controles.

**Resultados:**

os resultados mostraram que déficits nas medidas de VP podem ser indicadores precoces do declínio cognitivo nos casos de CCL, mesmo quando as funções executivas (FE) e a funcionalidade estão preservadas. Por outro lado, DA *versus* CCL mostrou diferenças em VP, EF e funcionalidade.

**Conclusões:**

As análises ROC mostraram que as medidas de PS tinham capacidades discriminativas para diferenciar CCL, DA e participantes controles.

## INTRODUCTION

Aging is usually referred to as a process of gradual deteriorations in the cognitive function that occur as people age. Profiles of cognitive aging include normal age-related cognitive declines from mild cognitive impairment (MCI) to a full stage of dementia.^1^ ^,^ ^2^ Alzheimer's disease (AD) is the most widespread form of dementia,^3^ and it is estimated that one in 85 individuals would be living with AD by the year 2050.^4^ Although the average duration of the disease varies between 4 and 8 years, some patients may survive up to 20 years with the disease.^5^ Similarly, the aging of the population leads to the prevalence of clinical conditions such as MCI.^6^ ^,^ ^7^ MCI is a clinical entity according to which individuals demonstrate cognitive deficit with minimal impairment of instrumental activities of daily living (IADL)^8^ ^,^ ^9^ and with substantial heterogeneity in etiology, clinical presentation, prognosis, and outcome.^10^ The prevalence of MCI varies according to variables such as clinical setting and inclusion criteria. However, this prevalence generally ranges from 11 to 20%.^11^ ^,^ ^12^ To better understand MCI has become a major public health priority. Its causes must be investigated, underlying pathophysiological processes and the earliest possible identification.^13^ Neuropsychological assessment has been effective in discriminating normal aging from mild cognitive impairment.^14^ ^,^ ^15^ Likewise, McKhann et al. reviewed the criteria for AD and found that clear-cut history of worsening of cognition by report or observation is one of the core clinical criteria for probable AD.^16^

Neuropsychological instruments are potentially non-invasive methods to identify individuals with MCI or predict the risk of developing MCI or dementia.^17^ Neuropsychological assessment is typically used for both descriptive and diagnostic purposes.^18^ When using the tests for diagnostic purposes, they provide information about the probability that an individual has — or will have at some moment in the future — to develop a cognitive disorder or deficit such as AD and MCI.^19^

The current literature recognizes neuropsychological heterogeneity in MCI by dividing it into subtypes. Most researchers employ four subtypes depending on the number of affected domains, namely: amnestic single-domain MCI (aMCI), amnestic multidomain MCI (aMCI), non-amnestic single-domain MCI (naMCI), and non-amnestic multidomain MCI (naMCI).^9^ ^,^ ^20^ ^–^ ^24^ The European Union (EU) report highlights the need to assess non-amnestic aspects in MCI, such as motor/perceptual aspects or processing speed (PS), considering that such features may not be thoroughly investigated and could represent early indicators of cognitive decline.^25^ Most neuropsychological studies involving MCI have focused on disorders of episodic memory, language, and executive functions.^26^ ^–^ ^31^ Actually, information processing speed is included in the diagnostic criteria for neurocognitive disorders of the Diagnostic and Statistical Manual of Mental Disorders (DSM-5).^16^ However, there is substantially less research that evaluates deficits in PS in MCI.^32^ ^–^ ^34^ Likewise, recent studies highlight the cognitive heterogeneity in AD, showing the importance of studying other cognitive aspects in addition to episodic memory.^35^ ^–^ ^38^

Normal aging and some psychiatric disorders (such as MCI and AD) were associated with decline in PS.^39^ ^,^ ^40^ PS involves several components of executive control, which vary according to age. Individual differences in PS indicate variation in neural speed,^41^ ^,^ ^42^ as well as age-related changes in neural processing, including the decline of axonal myelination throughout life.^40^ ^,^ ^41^ PS can be conceptualized as either the amount of time it takes to process a specific quantity of information^39^ ^,^ ^40^ or the quantity of information that can be processed within a finite amount of time.^43^ Decline in PS leads to cognitive deficits that make the ability to simultaneously control information limited. Taking into consideration the lower capacity to process information, it can also conduct to increased errors in the cognitive processing.^39^ ^,^ ^40^

Accurate diagnosis of MCI and AD is very important for timely therapy and possible delay of the disease.^44^ Consequently, the analysis of PS measures in aging is crucially important, especially in the Brazilian context, and neuropsychological tests are fundamental in this process. Based on a brief review of the literature, there are no Brazilian study on the subject to the best of our knowledge. Therefore, the purpose of this study is to analyze the diagnostic accuracy of early neuropsychological indicators, such PS measures, in older adults affected by AD, MCI, and in those who are cognitively-healthy.

## METHOD

### Participants

**Figure 1:**
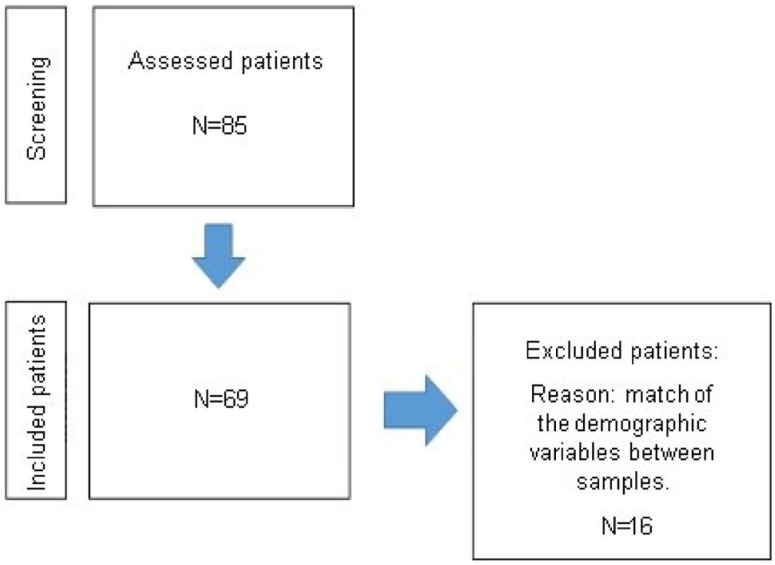
Flowchart of criteria for sample selection.

A total of 85 individuals were selected from a social program that was offered by the Government of Rio de Janeiro, Brazil. Of these individuals, 36 were control participants (CP), 26 had MCI, and 21 had a probable diagnosis of AD. The assessments were performed between 2016 and 2018 in Rio de Janeiro (state of Rio de Janeiro, Brazil) by a certified board psychiatrist and all neuropsychological evaluations were conducted by a senior neuropsychologist in Rio de Janeiro. The match of the variables “age” and “years of education” was performed; thus, 12 control participants and four individuals with MCI were excluded from the sample (Figure 1). Although the AD group presented a higher mean age, individuals with such diagnosis were not excluded, in such a way 21 AD cases of baseline were maintained. Therefore, the sample resulted in 26 CP and 22 individuals with MCI. All participants aged over 60 years and were proficient in Brazilian Portuguese. The participants agreed to participate in the study and signed the informed consent form. The study was approved by the Research Ethics Committee under authorization No. 965.264.

### Diagnosis

Control participants (CP) were individuals with no changes in cognitive performance tests and without functional impairment. The assessment of CP and individuals with MCI was based on clinical history, neuroimaging, and an initial neuropsychological protocol that included the following tests and scales: 1) Mini-Mental State Examination (MMSE);^45^ 2) Brief Cognitive Screening Battery, which consisted of the following tests: Memory of Figure Test (MFT); Categorical Verbal Fluency Test (CVFT); Clock Drawing Test (CDT); Geriatric Depression Scale (GDS-15); Pfeffer's Functional Activities Questionnaire (FAQ); and Lawton Instrumental Activities of Daily Living Scale (IADL);^46^ ^,^ ^47^ 3) Rey Auditory Verbal Learning Test (RAVLT);^48^ 4) Phonemic Verbal Fluency Test (FAS);^49^ and 5) Rey Complex Figure Task.^50^ All participants with MCI who had subjective cognitive complaints underwent a comprehensive neuropsychological assessment. A clinical diagnosis of individuals with MCI was established at a conference for each patient by an interdisciplinary team. Finally, the diagnosis of AD was based on the consensus criteria from the National Institute of Neurological and Communicative Disorders and Stroke and the Alzheimer's Disease and Related Disorders Association (NINCDS-ADRDA).^16^ Exclusion criteria included: 1) history of cerebral infection or stroke; 2) brain tumor; 3) head injury; 4) ongoing psychiatric illness; 5) history of alcohol or drug abuse; and 6) brain imaging that indicated any possibility of brain lesions other than MCI.

### Neuropsychological tests used in measures of processing speed and executive functions

PS was assessed by the tests of the Wechsler Adult Intelligence Scale, Third Edition (WAIS-III),^51^ namely: Digit Symbol-Coding (CD) and Symbol Search (SS). In addition, the Processing Speed Index (PSI) was performed for all participants in the sample. Finally, the attention and executive functions were assessed by the Color Trails Test (CTT),^52^ the Victoria Stroop Test (VST, Dot condition — Card 1; Word condition — Card 2; Interference condition — Card 3),^53^ and the Digit Span Test (DS).^51^

### Analyses

All analyses were conducted with the *Statistical Package for the Social Sciences* (SPSS) software, v. 21,, with significance set at p≤0.05. Normality of distribution was determined by a histogram. According to the data, there was no normal distribution, in such a way that parametric and nonparametric tests were performed. The results of the analyses only differed in CTT — Form A (individuals with MCI) and VST (Card 1; individuals with MCI). Measures of Skewness and Kurtosis were analyzed and indicated the use of parametric tests. Data analysis was performed using raw scores of the neuropsychological tests, and only PSI was converted into standard scores. The relationship between dependent variables and the clinical group was tested with analysis of variance (ANOVA). If a significant ANOVA was found, *post hoc* tests (Bonferroni test) controlling for multiple comparisons were used to identify pairs of clinical groups that significantly differed. Clinical groups were also compared according to demographic characteristics (i.e., age, sex, and years of education). Finally, the Receiver Operating Characteristic (ROC) curve was performed for the following tests and index: CD, SS, and PSI. ROC curves were also plotted in order to determine the degree to which subtests discriminated between CP, individuals with MCI, and individuals with AD. These analyses show the sensitivity *versus* one minus the specificity for each possible cutoff point. The area under the curve (AUC), with 95% confidence intervals (95%CI), was used as an indicator of the ability of the PS measures in differentiating patients who were CP, individuals with MCI, and individuals with AD.

## RESULTS

### Demographic characteristics, Mini-Mental State Examination, and Lawton scores

Table 1 summarizes demographic data, Lawton score (patient version), MMSE score, and pairwise comparisons. The repeated measures ANOVA showed differences between age (*F* (2.66)=5.7; p=.005); MMSE score (*F* (2.66)=21.5; p<.001), and Lawton score (*F* (2.65)=28.1; p<.001), but not in years of education (*F* (2.66)=2.5; p=.084). When comparing individuals with MCI *versus* CP, pairwise comparisons presented no differences in age (p=.56), years of education (p=.09), MMSE score (p=.22), and Lawton score (p=.66). As expected, AD *versus* MCI showed differences in MMSE scores (p<.001) and Lawton scores (p<.001), but not in age (p=.14) and years of education (p=1.00).

**Table 1:** Demographic characteristics (mean and standard deviation), Mini-Mental State Examination scores (maximum: 35 points), Lawton scores (patient version; maximum: 21 points), and pairwise comparisons for the three study groups.

	CP (n=26)	MCI (n=22)	AD (n=21)	F	*p-value	*p-value (CP versus MCI)	*p-value (MCI versus AD)
**Age**	73.3 (4.9)	75.6 (6.2)	79.2 (6.7)	5.7	**0.004**	0.568	0.147
**Years of education**	13.1 (3.0)	10.4 (5.1)	11.4 (4.5)	2.5	2.5	0.087	1.00
**MMSE score (max: 35 points)**	31.6 (1.8)	29.6 (2.6)	24.4 (5.9)	21.5	**<0.001**	0.225	**<0.001**
**Lawton score (max: 21 points)**	20.8 (0.4)	20.3 (0.6)	18.0 (2.2)	28.1	**<0.001**	0.665	**<0.001**
**Men**	4 (22)	1(21)	7 (14)	*	*	*	*

* MMSE: Mini-Mental State Examination; CP: Control Participants; MCI: mild cognitive impairment; AD: Alzheimer's disease.

### Executive functions and processing speed measures

Table 2 shows mean and standard deviation (SD) of neuropsychological measures and pairwise comparisons. The ANOVA demonstrated differences in PSI (*F* (2.66)=25.1; p<.001), SS score (*F* (2.66)=17.2; p<.001), CD score (*F* (2.66)=26.3; p<.001), SS errors (*F* (2.66)=3.6; p=.030), VST-Card 1 (*F* (2.57)=6.3; p=.003), VST-Card 2 (*F* (2.57)=6.6; p=.002), VST-Card 3 (*F* (2.57)=9.5; p<.001), CTT – Form A (*F* (2.66)=15.5; p<.001), CTT – Form B (*F* (2.66)=18.0; p<.001), CTT – Form A errors (*F* (2.66)=4.1; p=.021), and DS (*F* (2.66)=5,5; p=.006), but no differences in CTT — Form B errors (*F* (2.66)=3.0; p=.055). When comparing individuals with MCI and CP, only PS measures showed differences, such as: SS score (p=.001), CD score (p<.001), PSI (p<.001), and SS errors (p=.028). There were no differences in EFs measures, except for DS (p=.04). This ability to identify early MCI in individuals can be explained by differences in the performance of PS measures, which is sufficient to distinguish the groups. However, the comparison between MCI and AD demonstrated differences in PS and EFs measures, such as: CD score (p=.010), PSI (p=.036), CTT — Form A (p=.002), CTT — Form B (p<.001), VST-Card 2 (p=.012), and VST-Card 3 (p=.002).

**Table 2:** Mean and standard deviation of neuropsychological measures and pairwise comparisons.

Neuropsychological measures	CP (n=26)	MCI (n=22)	AD (n=21)	*p-value (CP versus MCI)	*p-value (MCI versus AD)
**PSI (max: 146 points)**	123.0 (12.5)	107.1 (11.2)	97.1 (14.2)	**<0.001**	**0.036**
**CD (max: 133 points)**	45.7 (13.7)	30.7 (11.5)	18.8 (12.7)	**<0.001**	**0.010**
**SS (max: 60 points)**	24.8 (9.9)	15.5 (6.1)	11.2 (7.5)	**0.001**	0.277
**SS errors**	1.5 (1.5)	3.0 (1.7)	2.4 (2.2)	**0.028**	0.931
**CTT – Form A**	67.0 (27.0)	94.9 (27.6)	153.9 (88.3)	0.231	**0.002**
**CTT – Form B**	130.9 (45.2)	170.3 (38.3)	266.2 (126.2)	0.257	**<0.001**
**CTT – Form A errors**	0.04 (0.1)	0.14 (0.4)	0.6 (1.2)	1.00	0.097
**CTT – Form B errors**	0.15 (0.3)	0.64 (1.0)	1.9 (4.3)	1.00	0.291
**VST-Card 1**	14.8 (3.6)	21.7 (8.4)	32.3 (25.0)	0.506	0.095
**VST-Card 2**	21.0 (6.3)	24.7 (7.3)	53.2 (51.6)	1.00	**0.012**
**VST-Card 3**	33.6 (13.4)	40.2 (12.8)	87.3 (70.8)	1.00	0.**002**
**Interference**	2.0 (0.9)	2.5 (2.3)	2.7 (1.1)	1.00	0.05
**DS (max: 14 points)**	12.4 (3.7)	10.1 (2.6)	9.6 (2.7)	**0.04**	1.00

* VST: Victoria Stroop Test; CTT: Color Trails Test; SS: Search Symbols (maximum: 60 points); CD: Digit Symbol-Coding (maximum: 133 points); PSI: Processing Speed Index (maimum: 146); DS: Digit Span Test (maximum: 14 points).

### Mild cognitive impairment *versus* control participants

**Figure 2:**
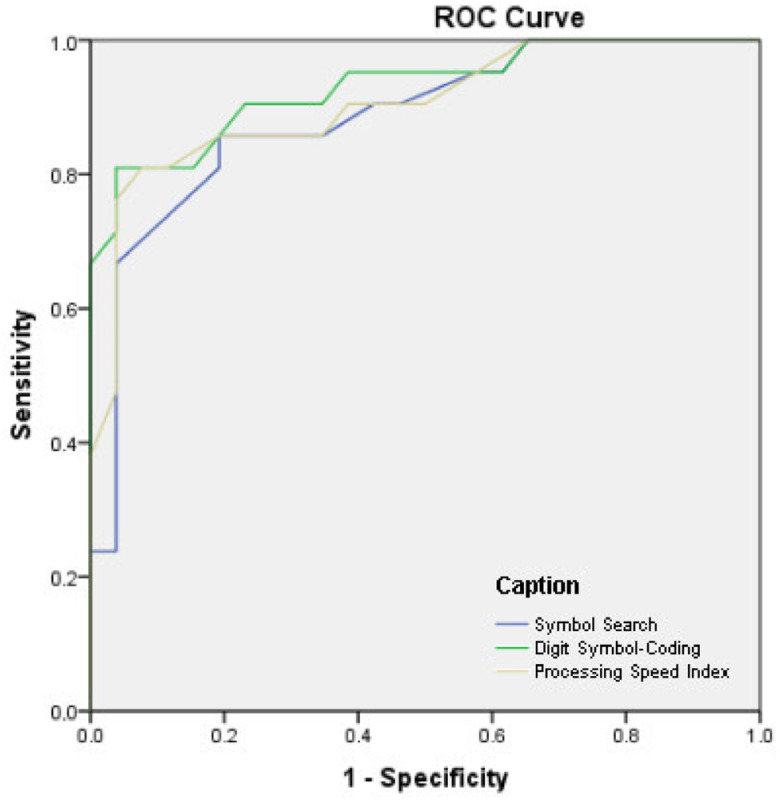
Analyses of Receiver Operating Characteristic curve, sensitivity, and specificity for Alzheimer's disease *versus* control participants.

Sensitivity and specificity of the processing speed measures were investigated in the first analysis, namely: SS, CD, and PSI. These diagnostic parameters were used to test the ability of these PS measures in identifying individuals with MCI compared with cognitively-healthy older adults (Figure 2). The estimated AUC for SS was 0.80 (95%CI 0.68–0.93; p<0.01) and for CD, 0.79 (95%CI 0.66–0.92; p<0.01). In addition, the estimated AUC for PSI was 0.83 (95%CI 0.71–0.94; p<0.01). The most appropriate cutoff point (raw score) for SS was 19.5, with sensitivity and specificity of 86 and 76% respectively; as for CD (raw score), the most appropriate cutoff point was 36.5, with sensitivity and specificity of 77 and 80% respectively. Finally, the most appropriate cutoff point for PSI was 114.5, with sensitivity and specificity of 81 and 76% respectively.

### Alzheimer's disease *versus* control participants

**Figure 3:**
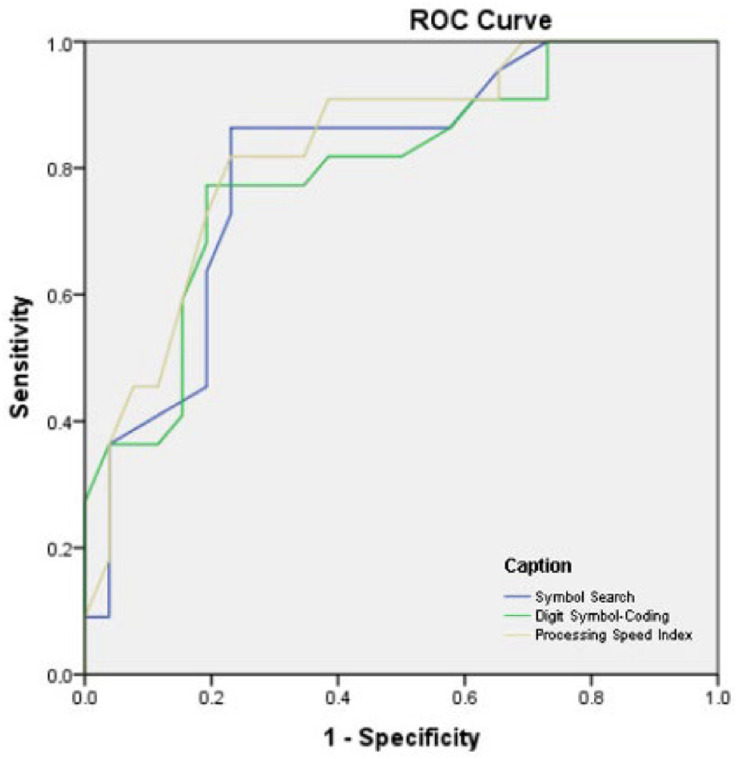
Analyses of Receiver Operating Characteristic curve, sensitivity, and specificity for individuals with mild cognitive impairment *versus* control participants.

The diagnostic parameters were used to test the ability of PS measures in identifying cases of Alzheimer's disease compared with cognitively-healthy older adults. The estimated AUC compared with cognitively-healthy older adults. The estimated AUC (Figure 3) for SS was 0.88 (95%CI 0.78–0.98; p<0.01); for CD, 0.92 (95%CI 0.85–0.99; p<0.01). Finally, the estimated AUC for PSI was 0.90 (95%CI 0.81–0.99; p<0.01). The most appropriate cutoff point for SS (raw score) was 17.0, with sensitivity and specificity of 85 and 80% respectively; as for CD (raw score), the most appropriate cutoff point was 35.5, with sensitivity and specificity of 85 and 80% respectively. Finally, the most appropriate cutoff point for PSI was 112, with sensitivity and specificity of 85 and 80% respectively.

## DISCUSSION

The results of this study might indicate that individuals with MCI could be early identified according to the performance in PSI and in tests that assess PS, when compared with the control participants. According to Salthouse,^39^ ^,^ ^40^ the decrease in PS can also lead to increased errors in the cognitive processing. Moreover, according to data of the present study, samples of individuals with MCI and CP can be differentiated by the number of errors made in one of the PS tests. However, differences in traditional measures of executive functions were not enough to distinguish control participants from individuals with MCI, except for DS. This fact can be explained because complex measures of EFs, such as working memory, require process of attention and mental manipulation.^54^ Furthermore, the ROC analyses showed that PS measures had discriminative capacities to differentiate individuals with MCI, AD, and cognitively-healthy older adults.

Cognitive domains decrease with advanced age.^55^ ^,^ ^56^ Decline in cognitive function affects more than 50% of people aged over 60 years.^57^ Particularly, memory and PS seem to be more sensitive to age than other cognitive domains.^39^ ^,^ ^55^ A recent study have compared cognitively-healthy older adults, individuals with MCI, and individuals with AD, and showed that PS measures were significant to differentiate the three groups. White matter (WM) brain pathology is often present in patients with MCI and AD. Thus, this study concluded that WM seemed to have the strongest effects on PS measures for the three samples.^33^ Likewise, Park et al. assessed cognitively-healthy older adults, individuals with MCI, and those with AD, and concluded that a PS measure could distinguish the three groups.^34^ These results support our findings and the notion that neuropsychological measures are sensitive to differentiate individuals with AD, MCI, and cognitively-healthy older adults.

According to the American Psychiatric Association (APA) and the NINCDS‐ADRDA, neuropsychological assessment is necessary and consists in an important component for the diagnosis of MCI and AD, respectively.^16^ ^,^ ^58^ Furthermore, neuropsychological testing is an equally valuable and arguably more affordable and less invasive cognitive biomarker of AD.^20^ In this context, the strength of the study was to provide diagnostic parameters for early neuropsychological indicators in the Brazilian samples. In addition, it provides raw data on the performance of individuals with MCI compared with cognitively-healthy older adults.

However, limitations of this study should be discussed. First, the Brazilian norms of the WAIS-III have limitations and must be revised. In order to minimize these biases, only the raw data from the CD and SS tests were analyzed. Second, the study sample size. Clinical samples and extensive neuropsychological evaluations present major obstacles to evidence-based neuropsychology practice. Noteworthily, the sample of the present study is mainly composed of women. Nevertheless, previous studies showed that women have better performance than men on verbal memory tasks, but sex differences were not evident for speed of information processing and attention.^59^ ^,^ ^60^

In conclusion, the authors emphasize the importance of early indicators of cognitive decline in MCI and the diagnostic parameters in the neuropsychological instruments in the Brazilian clinical settings. Such aspects might impact the prognosis of the disorder and assist in decision-making concerning treatment options, especially those related to cognitive rehabilitation. Nevertheless, further studies on the subject are still necessary.
